# Women's experiences with the post-placental intrauterine device: a qualitative study

**DOI:** 10.61622/rbgo/2024rbgo45

**Published:** 2024-05-27

**Authors:** Ana Beatriz Venturin, Debora Bicudo Faria-Schützer, Odette del Risco Sánchez, Larissa Rodrigues, Thuany Bento Herculano, Fernanda Garanhani Surita

**Affiliations:** 1 Universidade Estadual de Campinas Department of Obstetrics and Gynecology Campinas SP Brazil Department of Obstetrics and Gynecology, Universidade Estadual de Campinas, Campinas, SP, Brazil.

**Keywords:** Intrauterine devices, Postpartum period, Long-acting reversible contraception, Reproductive health

## Abstract

**Objective:**

To explore women's experiences with postpartum intrauterine device (PPIUD) insertion and the decision-making process in the postpartum period.

**Methods:**

A qualitative design was employed with face-to-face interviews using a semi-structured script of open questions. The sample was intentionally selected using the concept of theoretical information saturation.

**Results:**

Interviews were conducted (1) in the immediate postpartum period, and (2) in the postpartum appointment. 25 women (N = 25) over 18 years old who had a birth followed by PPIUD insertion were interviewed between October 2021 and June 2022. Three categories were constructed: (1) Choice process, (2) Relationship with the health team at the time of birth and the postpartum period, and (3) To know or not to know about contraception, that is the question.

**Conclusion:**

Professionals’ communication management, popular knowledge, advantages of the PPIUD and the moment PPIUD is offered play a fundamental role in the construction of knowledge about the IUD. Choice process did not end in the insertion.

## Introduction

The intrauterine device (IUD) is a long-acting contraceptive method (LARC) with proven high effectiveness. The IUD failure rate is less than 1% during the first year of use.^(1,2)^ Worldwide, the percentage of IUD use is 16.8%, ranging from 6.5% in high-income countries to 3% in low-income countries.^(2)^ Data from the Brazilian Health Ministry showed that 1.9% of Brazilian women of reproductive age use the copper IUD despite it being a free method provided by the Unified Health System.^(3,4)^

Obstacles to adherence to the IUD include a lack of information for women about the mechanism of action, side effects and IUD effectiveness. In addition, a lack of trained professionals for its insertion, strict protocols that require performing several tests before insertion and frequent trips to the health service are challenges to IUD adherence.^(5,6)^

Insertion of the IUD in the immediate postpartum period is also called a post-placental IUD (PPIUD). Despite presenting a higher expulsion rate than the interval insertion, the cost-benefit analysis data endorse the superiority of immediate placement in reducing unplanned pregnancies, particularly for vulnerable women who are more likely to miss the postpartum appointment.^(7,8)^

When women have autonomy in choosing contraception, they feel satisfied when they are heard or when their individual wishes are met. Qualitative research shows the perspective of women on the promotion, accessibility and acceptability of LARCs. In this context, qualitative research can broaden the understanding of the lived experience, giving women a voice on reproductive issues. Additionally, healthcare teams can be instrumentalised in a naturalistic way for a humanising approach.^(9,10)^

Therefore, the objective of this study aims to explore women's narratives on PPIUD insertion and revisit the decision-making process about contraception in the immediate postpartum period.

## Methods

This is a qualitative study with face-to-face interviews following a semi-structured script of open questions conducted in the immediate postpartum period and the postpartum appointment. The qualitative research makes lived experiences comprehensible through the meanings attributed by the participants themselves in their experiences. In this method, individual interviews are considered a research tool that seeks to interpret in depth the descriptions of the lived experiences in the health context.^(11)^

The research was conducted at the Women's Hospital from the University of Campinas, Brazil – a tertiary teaching hospital specialising in women's health in the public health system.

Women over 18 years old who gave birth at the Women's Hospital followed by PPIUD insertion were included. The construction of the sample was carried out for convenience considering the criterion of theoretical saturation in which the researchers identified main patterns of relevant frequent and common meanings among the interviews. These patterns were organised into categories and subcategories.^(12)^

Initially, the main researcher performed a phase of acculturation in the rooming-in at the Women's Hospital between July and September 2021. This phase consisted of moments in which the researcher inserted herself in the environment in which the research was conducted and observed the relationships between service users and healthcare professionals in addition to observing procedures and working relationships. Information from this phase of the study was recorded in a field diary and was used to refine the questions initially proposed for the interviews.

Participants were invited into the study during the immediate postpartum period while they were in rooming-in, within the first 48–72 h after birth. The first author accessed the medical records of the service users and invited all women who had inserted PPIUD.

The volunteers’ approach included the following moments: (a) mutual presentation between researcher and interviewee with an exposition of the objectives of the interview, explanation and signatures of the informed consent form; (b) collection of sociodemographic data whose application benefited establishing a rapport; (c) a semi-structured interview with a script with open questions, encouraging the interviewee to talk about the subject of the study and ensuring respect for the interviewee's statements; and (d) completion of the interview, where the interviewer asked the interviewee if she wanted to complement or add something that was not previously mentioned.

**Figure 1:**
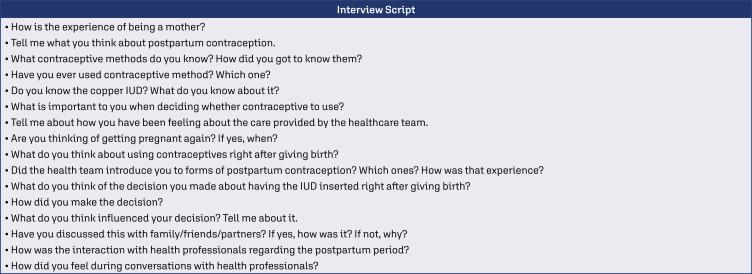
Interview script

The interviews were carried out in two moments with each participant: (1) before hospital discharge, and (2) at the postpartum appointment, between 40 and 60 days after birth, both moments in a silent and private space. All interviews were recorded in audio and transcribed in full for later analysis (Figure 1).

Six meetings were held between May 2022 and April 2023 to address the first 6 of 7 steps planned for Clinical-Qualitative Content Analysis.

All interviews conducted during the immediate postpartum and postpartum appointments were analysed as a set. For examination of the data, a thematic analysis was used following the seven steps provided for Clinical-Qualitative Content Analysis.^(13,14)^ The clinical-qualitative analysis involves a critical reflection on the processes carried out at each stage with other qualitative researchers, which is why steps 1 to 4 of the analysis were carried out by three authors and steps 4 and 5 involved another author of the article to refine and broaden the understanding of the results.^(14)^

Validation of the results was carried out in two meetings with the SARHAS (Reproductive Health and Healthy Habits) Research Group composed of a multidisciplinary team of researchers. Moreover, partial results were presented at the 2022 Brazilian Congress of Gynecology and Obstetrics.^(15)^ The Consolidated Criteria for Reporting Qualitative Research checklist was also used to ensure the qualitative rigour of the study.^(16)^

This study was approved by the Research Ethics Committee of the State University of Campinas (UNICAMP) under number 4.956.066/ *Certificado de Apresentação de Apreciação Ética:* 50670921.9.0000.5404.

## Results

Twenty-five women were interviewed between October 2021 and June 2022. The participants were between 18 and 42 years old. All subjects had prenatal care (number of appointments self-declared by participants) and most completed high school and lived with a partner, as described in table 1.

**Table 1:** Characteristics of the interviewees

# Interviewee	Age (years)	Skin color	Schooling	Partner	Postpartum review interview
1	30	White	High School	Yes	Yes
2	42	White	Elementary School	No	No
3	21	Mixed	High School	No	Yes
4	34	Mixed	High School	Yes	Yes
5	20	Mixed	High School	Yes	Yes
6	25	Mixed	High School	No	No
7	25	Mixed	High School	Yes	Yes
8	24	Mixed	High School	Yes	Yes
9	32	White	High School	Yes	No
10	19	Black	High School	No	No
11	21	White	High School	Yes	Yes
12	35	Black	High School	No	Yes
13	35	White	High School	Yes	No
14	32	Mixed	High School	Yes	Yes
15	21	White	Elementary School	No	No
16	23	Black	Elementary School	No	Yes
17	34	Mixed	High School	Yes	No
18	37	White	Higher Education	Yes	No
19	26	Black	High School	Yes	No
20	18	Mixed	Elementary School	No	No
21	42	White	Higher Education	Yes	No
22	25	Mixed	Elementary School	No	No
23	27	Black	High School	Yes	Yes
24	20	White	Incomplete Elementary School	No	No
25	26	White	High School	Yes	No

**Figure 2:**
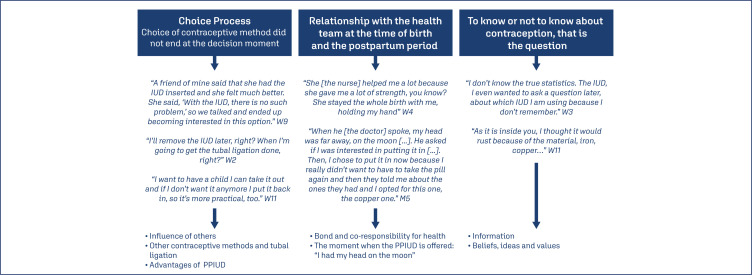
Comprehending interview reports and the category construction process

During content analysis, 3 categories and 7 subcategories were constructed as described in figure 2.

### Choice process

The choice of contraceptive method comprises a dynamic process involving psychosocial and intrapsychic elaborations. The choice of contraceptive method does not end at the moment of decision or in the act of choosing. Therefore, we must understand the choice as a complex process that cannot be isolated in time. This process is influenced by psychosocial aspects constructed during the women's experiences related to contraception during their reproductive life.

### Influence of others

Women resorted to others’ experiences and opinions about the IUD to make their decision about contraceptive use. The people mentioned are close figures who had a place of affection in the women's lives, as shown in the following statements:

*In fact, a friend of mine said that she had the IUD inserted and she felt much better. She said, ‘Instead of taking the pill, taking an injection that can cause some other reaction, go after the IUD because you don't have that concern: "Oh, I'm going to fail, then I failed to take the medicine, I forgot."’ She said, ‘With the IUD, there is no such problem,’ so we talked and ended up becoming interested in this option. (W9)*

*There were me and my mother in the room [during childbirth], then my mother who used the IUD for a long time, she said: ‘Put it in because I used it for a long time and it's very good.’ Then, I said, ‘So, ‘okay.’ She said it was good, so I said [to the healthcare professionals], ‘Then you can put it in.’ (W12)*

A kinship was sometimes unnecessary for the opinion of the close person to be relevant to the woman. What counted was the affection and reference that the people involved had in the woman's life.

*Even my social worker also uses the IUD. She advised me a lot. I also looked a lot for references on the internet; I tried to inform myself a little more… And that was it. […] She is an excellent social worker, you know? She takes great care of us here. And she advised me a lot, she gave me a lot of confidence because she uses the same IUD, she uses the copper one too. (W14)*

### Other contraceptive methods

Decisions to use the IUD were based on experience with other contraceptive methods and the desire for something definitive and practical. Women often said they arrived at the health facility wanting to have a tubal ligation procedure. In addition, they said that they did not feel comfortable using other contraceptive methods, such as contraceptive pills and injections, due to side effects and the need to reapply doses periodically. Thus, the women in this study were more likely to use the IUD. There were cases of women who accepted the use of the IUD until they could undergo a tubal ligation procedure, as shown in the following statement:

*‘I'll remove the IUD later, right? When I'm going to get the tubal ligation done, right?’ [W2]*

Others commented on their previous experiences with contraception and the possibility of trying something new:

*‘I chose it because I don't trust the pill anymore and I don't trust the injection, either, so I chose the IUD.’ (W5)*

Side effects of hormonal contraceptive methods were cited as reasons for wanting to use the IUD:

*But I was always very afraid because I always read about thrombosis – about the risks of contraception in some cases because of the hormone. So I always felt very insecure and then I ended up choosing not to protect myself with hormones, only with a condom or there following the table. Because I was very afraid of having some… Thrombosis, a side effect. (W7)*

### Advantages of ppiud

Some points were raised regarding what was advantageous and practical in using the IUD and inserting PPIUD (Chart 1).

**Chart 1:** Advantages of postpartum intrauterine device (PPIUD) from women's perspectives

Advantages of PPIUD	Participants’ quotes
Practicality of the insertion	*At the time of birth, I could do everything at once*. (W1) *Perfect because I was already there in that emotion [birth/immediate postpartum], in that whole thing and this neighbour of mine said that when she went to put it in, it bothered a little bit; I really felt that discomfort. But for those who were already in the rain, I said: ‘No, let's get wet for good! We're going to put it in now’. So, I took advantage of the moment to put it in*. (W9)
Accessibility to healthcare services and IUD free access	*The difference is that our cervix is more open, I think it is, right? That's the difference. Oh, it's the benefit, you know, that I'm already here… I don't have to come here again… Ah, because we have easy access in this postpartum period, and it's free… And you don't have to keep reminding yourself of ‘Oh, you have to take something.’* (W21)
Reversible method	*I thought it was good to put it in after childbirth […]. Because you don't need to remember it every time to change it, and if I want to have a child I can take it out and if I don't want it anymore I put it back in, so it's more practical, too.* (W11)
Safety and long-lasting method	*Regarding my daily activities… The fact that I don't forget to take it or to inject it, to go to the pharmacy so I can have the injection. That's what I mean by safest. It's the lack of concern and the daily activities that sometimes I end up forgetting.* (W7)
Non-interference during the breastfeeding period	*Talking to my doctor, she gave me the option of this IUD. Then I talked to him [husband] and he agreed because the IUD would not harm breastfeeding for the baby. Because, at the moment, we saw it safer, apart from the condom, to protect ourselves in terms of being able to breastfeed her [baby].* (W18)

### Relationship with the healthcare team at the time of birth and postpartum period

The relationship with the healthcare team was described as generally good during birth and the postpartum period. Women were sometimes more specific about care regarding the IUD and PPIUD insertion.

### Bond and co-responsibility for health

Affection and trust in the healthcare professionals came in the form of body contact and non-verbal communication:

*She [the nurse] helped me a lot because she gave me a lot of strength, you know? She stayed the whole birth with me, holding my hand […]. So, when I got here the nurse came talking to me, so I thought that was very important because, for me, who wasn't even thinking about the method at that moment, it was very good. So, it was a very quick, practical solution, which I think will help a lot of people. (W4)*

Respect for the woman's body appeared when healthcare professionals communicated what they were doing:

*Then I thought it was good because they were explaining everything –how they were going to put it in. I already know how to insert (laughs). They explained what they were going to do, how they were going to do it, so that gave us more ownership because we're lying down, we don't know what's going on down there. (W11)*

### The moment when the ppiud is offered: ‘I had my head on the moon’

The results showed that the moment when the contraceptive method is offered generates different reactions among women in terms of the perception of choosing the method, cognition and the time involved in making the decision.

### Prenatal method offering

When the contraceptive method was offered during prenatal care, women's experiences included satisfaction with care and a desire for the method. Unlike the other participants, a prenatal care offering did not affect cognition or memory at the time of birth.

*Oh, I thought it was cool. Because even if that was her [physician's] intention [talking about contraception], I thought she was very… not direct… She knew how to approach the subject without saying ‘It's not for you to get pregnant now.’ (W25)*

Some women were convinced about the decision on the day of childbirth:

*And it was written on my medical report that I wanted the IUD. But as soon as I entered the room, I said, ‘Oh, I have the IUD to put in, huh?’ The nurses running took the IUD: ‘No, it's already here.’ I made them remember. (W16)*

### Offering the method at the time of childbirth

Women repeatedly said that healthcare professionals offered the IUD on the day of birth/during birth/immediate postpartum and about the level of awareness and attention at the time: ‘with my head on the moon’. That is, the women considered that they were not very attentive to what the health professional said when offering the method, as described in the statements:

*When he [the doctor] spoke, my head was far away, on the moon […]. He asked if I was interested in putting it in […]. Then, I chose to put it in now because I really didn't want to have to take the pill again and then they told me about the ones they had and I opted for this one, the copper one. […] It was very interesting… Because I already wanted someone to have talked to me about it, but I thought it would only happen after the postpartum appointment. Talking in that moment was good. (W5)*

*I was so nervous [in childbirth] that I wanted it to be over soon, so I didn't even ask. But since I have postpartum appointments, I'll come back and ask questions about the IUD. Clear my doubts. […] Their patience, the understanding they have for patients, equality… They treat each patient the same, too. I think everything the doctors do here is very good. (W20)*

Some women mentioned that they already wanted to use some method or were interested in the IUD before the device was offered by doctors:

*Then she [nurse] said: ‘Did you know that you can put it in the postpartum?’ I said: ‘No.’ Then she said, ‘If you want to put it in, we'll try to put it in.’ I said, ‘Great idea!’ I didn't even ask my husband, I said, ‘I want it!’ That was it, like, there was no time for me to think, the answer was very quick, but I liked it a lot. (W4)*

*I was very lucid, aware of what was going on. When he asked, I immediately replied ‘Yes,’ because I already had this thought of not wanting to have children anymore. So, for me it was super peaceful. (W19)*

### To know or not to know about contraception, that is the question

During the interviews, women answered that they knew nothing about the IUD. Although the lack of information was something very recurrent, many times the ‘knowledge’ that the women spoke about ignored all the popular knowledge they possessed. Possibly, when asked about their experience with the method by a researcher linked to the university, they felt intimidated and ended up relating ‘knowledge’ to ‘technical-scientific knowledge’. This observation allows us to assume that this experience is also experienced in the doctor–patient relationship.

### Information

Women referred to their own experience with the IUD when they said they knew nothing about the method. At other times, information about how the IUD works, IUD material, follow-up and how to insert it was really lacking. The women even associated the researcher with a doctor/nurse who could clarify their doubts during the interview. Participants mentioned how doctors and nurses talked about the IUD when they offered the method, but they could not remember what was said.

*The percentage of chances that it will… may or may not happen [pregnancy]… I don't know the true statistics. The IUD, I even wanted to ask a question later, about which IUD I am using because I don't remember. (W3)*

On the other hand, women talked about how the information provided by the healthcare professionals made her feel safer after the IUD was expelled:

*I already ‘knew’ [IUD], between quotation marks, that there was a copper IUD, a hormonal one, but I had never had contact. And then, when it came out [body expelled], I also looked at it and everything. I saw it and the girls [nurses] explained to me a lot about how it works and everything. Then, it was safer for me, too. (W7)*

Participants also spoke about misinformation conveyed by doctors and healthcare professionals from other health services about the insertion of PPIUD:

*My doctor said that IUDs should not be put in after childbirth because the uterus is swollen, and automatically, when the uterus is normal, it will expel the IUD. I don't know if this is true or not, so it was good to share this information because I don't know if I was the first one [for whom] the IUD didn't work out or if several people had it before me, in the same process that I had, and also it didn't work out with them. (W12)*

### Beliefs, ideas, and values

The ideas described by the women about the IUD were constructed from experiences from close people and individual beliefs. Many women demonstrated not having enough information or feeling confused when discussing the contraceptive method. Women believed that the IUD did not last for 10 years because of the copper material present in the device. A participant associated copper with iron, with the possibility of rusting over time.

*But I didn't know that it lasted 10 years, that I didn't need to change it. I thought that every time I needed to change it, you know. Because as it is made of iron, you know, copper, I thought it had to be changed, right? Because they say it rusts. I thought I had to change it, but now she [a health professional] told me that it goes up to 10 years […]. As it is inside you, I thought it would rust because of the material, iron, copper… Then I thought, ‘Wow, have you thought about putting it in there and rusting it?’ Then I got scared, but then she [nurse] told me that it doesn't rust. (W11)*

Many participants brought questions related to Brazilian news in which the media exposed photos of newborn children with the IUD in their hand/stuck in their hair: *'I've heard that the IUD moved, that the child was born with the IUD in hand, that's all these stories.’* (W13)

*I've seen on Facebook that the baby was born with an IUD wrapped in her hair. Is this true? The IUD has a way to come loose, right? […] So, in the case of this woman, I think the IUD came loose and she ended up getting pregnant. (W20)*

There were reports about beliefs created to fulfil their doubts:

*It's because we don't put something inside ourselves that we don't know what it means, what's going to happen, what's going to work… I found out that the IUD goes wrong, that the child is born with the IUD in his hand. These things […] I had doubts about putting the IUD in because I was afraid my uterus wouldn't accept it, because of the surgery I had done. I was afraid because I thought the IUD would not have any effect […]. My uterus is turned around. I thought that, because it is turned around, it wouldn't accept the IUD. It wasn't even because the doctor said ‘your uterus is turned around, it won't work out.’ I put the idea in my head and believed it. (W16)*

### The importance of welcoming women's beliefs, ideas, and values

Often the healthcare professional communicated to the woman everything she should know about the IUD before insertion. However, the woman for several reasons could not assimilate the information. In this way, women brought the experience of inserting the IUD as a moment of submission to medical knowledge and not as an active person in the process. The relationship between healthcare professionals and service users showed up in the interviews through the fear of approaching doctors and nurses to clarify doubts and ask for guidance. The interviewees said that they did not clarify their doubts when the professional offered the IUD. Women felt they could be ‘offended’ by the doctors if they showed their doubts about contraception or did something wrong from the doctor's point of view.

*Then they put it [the IUD] in. I don't know how they put it in because my leg was numb. I don't know if it was through the caesarean section […]. [About asking the doctor for guidance] Because otherwise, they [doctors] would discuss with me [about] taking medicine without being told to… They would offend me because I took medicine without them telling me to, without the doctor's guidance. My sister used to take [the contraceptive pill] to stop it [menstruation], she got cancer, passed away… People said she had cancer because of that, because of the medicine she took [without prescription]… (W23)*

A narrative focused on the difficulty of discussing contraception with doctors during pregnancy due to the idea that the baby, inside the womb, would feel rejected by the mother.

*You don't want to say ‘I don't want to have a child anymore,’ mainly because… That's what I think… I'm afraid of him [baby] feeling rejected when I talk about this subject in front of him, that's my thought, my vision. However, when I found out I was pregnant, as much as I didn't expect to be pregnant, I took it in stride because I knew that if I rejected him, he would be aware that I was rejecting him. (W19)*

## Discussion

Women's experiences with the use of the IUD in the immediate postpartum period involve revisiting their own experiences with the use of other contraceptive methods, other people's experiences with the IUD, the bond with the health professional who offers the method and the moment of offering the method.

Findings of this study are also consistent with literature stating that other interpersonal influences contribute to the contraceptive decision and how the family, for example, exerts a strong influence on whether to continue the PPIUD.^(17)^ In this sense, present data show that the experience, both of the woman herself and of close people, should be investigated in prenatal care and family planning appointments. The literature already describes that reproductive autonomy also has an impact on choosing more effective methods, such as LARCs.^(18)^

Advantages of PPIUD were presented as key elements in women's discourse during the choice process and the findings are like those found in the literature in which women feel satisfied and enthusiastic about the convenience of insertion right after childbirth.^(9,19)^

Receiving contraceptive guidance and tools for reproductive planning while still pregnant allows women to make autonomous and conscious decisions in the postpartum period.^(20,21)^ Hearing about the IUD while still pregnant increases the chance of accepting it in the postpartum period. The postpartum period is a psychologically vulnerable time for women and the discussion about contraceptive methods at this time may not be well assimilated and introjected.^(22,23)^

On the other hand, results of this study showed that women often described receiving the PPIUD insertion offer on the day of childbirth as a good opportunity. Considering how to offer contraceptive methods in the immediate postpartum period to those women who did not have access to contraceptive guidance during prenatal care is a more than necessary care, as it may represent the only opportunity for contraception for many women with limited access to healthcare services.

Therefore, it is necessary to consider ways to follow up with women during the postpartum period to reassess the decision to insert the IUD, as it is not a choice that ends with insertion but rather a process that remains even after choosing the method.^(24)^ For example, there were cases in which women decided to reinsert the IUD in the postpartum appointment and were able to understand more about the device in this second meeting with healthcare professionals.

According to the Brazilian National Primary Care Policy, bonding ‘consists of building relationships of affection and trust between the user and the health worker, allowing the deepening of the process of co-responsibility for health, built over time".^(25)^ From this perspective, it is in the relationship that there will be a space for listening to the information that women already have about the IUD and being able to argue about what science says so that the woman can make the decision. Therefore, the embracement and active listening of healthcare professionals are linked to satisfaction with care and bonding.^(9)^

The results of this study are in line with other qualitative studies reinforcing the importance of personal experiences, beliefs and values in choosing how to exercise sexual and reproductive rights.^(18,26,27)^ Findings showed that the participants had beliefs and ideas about the IUD, such as the idea of the device rusting, not fitting in the uterus, and mentioning in the prenatal period the desire to use some contraceptive method as a rejection of the unborn child. Brazilian IUD news were mentioned a lot by women during the interviews and this fact is a strong representation of reproductive education and access to healthcare in Brazil.

Despite being distributed by the Brazilian Public Health System, many barriers are encountered in accessing IUD. Statements may be linked to the lack of discussion and education about contraceptive methods in formal education, taboos related to the topic, and the lack of access to IUDs in healthcare services.

People tend to build knowledge about the method through popular knowledge that sometimes can be disregarded by healthcare professionals. Alspaugh et al. (2020)^(26)^ found similar results when discussing communication and power relations between healthcare professionals and health service users. The lack of acceptance of this knowledge can lead to the submission of women to medical knowledge since they understand that only this knowledge is valid. Submission was manifested when women did not even consider taking their doubts to the doctors or when they openly said that they felt that the professionals would repress them if they did. In this way, hearing about the meanings attributed to contraceptives can provide important elements for the discussion on contraception.

The study showed that there is something beyond what is said during the questionnaires of quantitative studies that are impossible to capture without an open interview. Participants’ beliefs and ideas could only appear in the face of open questions and patient listening. Nevertheless, this work was limited to the responses of women from a specific and referenced health service in the region. Some issues may not have been mentioned by the interviewees due to the environment in which the interview was carried out and a possible fear that the interviewer was part of the health team and consequently did not offer the most comfortable conditions that would allow the woman to make any criticism to the service.

Healthcare professionals often advise on contraceptive methods, but what the woman hears may be different from what the professional says. The action of listening and accepting the women's beliefs and ideals without judgment is a way of horizontalising the relationship and creating a feeling of security and satisfaction in service users. Affection not only with people close to the woman but also with healthcare professionals is considered a crucial aspect in the process of choosing the contraceptive method. Family, friends, partners and the history of their experiences often determine what the woman will choose.

## Conclusion

The results of this study reinforce the importance of listening to women about their experiences and those around them about contraceptive methods. The professionals’ communication management and popular knowledge play a fundamental role in the construction of knowledge about the IUD. The women mentioned the advantages of the IUD as vital elements in their choice and discussed how information about the device could be assimilated when they were given the IUD during birth/immediate postpartum. In this sense, this study reinforces that the ideal time to offer and discuss contraceptive methods in the immediate postpartum period is antenatal care or earlier moments during women's reproductive planning. When this cannot be done during ANC, offering PPIUD in a moment close to birth could be an option with some caution regarding the vulnerability of the moment. In addition to respecting the woman's history, inviting narratives into the health services can open space for debate regarding myths about contraceptive methods. When healthcare professionals listen, they have a starting point to initiate a conversation about contraception.
